# Tumor suppression, dose-limiting toxicity and wellbeing with the fetal estrogen estetrol in patients with advanced breast cancer

**DOI:** 10.1007/s00432-020-03472-8

**Published:** 2020-11-26

**Authors:** Marcus Schmidt, Hans Lenhard, Arnd Hoenig, Yvette Zimmerman, Jan Krijgh, Monique Jansen, Herjan J. T. Coelingh Bennink

**Affiliations:** 1 Department of Obstetrics and Gynecology, University Medical Center Mainz, 55122 Mainz, Germany; 2 Department of Obstetrics and Gynecology, Katholisches Klinikum Mainz, 55131 Mainz, Germany; 3 Pantarhei Oncology BV, Boulevard 17, 3707 BK Zeist, The Netherlands

**Keywords:** Advanced breast cancer, Estetrol (E4), High-dose estrogen (HDE) treatment

## Abstract

**Purpose:**

The aim of this study (the ABCE4 study) was to assess dose-limiting toxicity (DLT), safety, tolerability and preliminary efficacy of high doses of the fetal estrogen estetrol (E4) in postmenopausal patients with heavily pretreated, locally advanced and/or metastatic ER+/HER2−breast cancer, resistant to anti-estrogens.

**Methods:**

This was a multicenter, open-label, phase IB/IIA, dose-escalation study with a 3 + 3 cohort design, whereby successive cohorts of three patients received 20 mg, 40 mg or 60 mg E4 per day for 12 weeks by oral administration. DLTs, safety and wellbeing were evaluated after 4, 8 and 12 weeks of treatment. Anti-tumor effects were investigated by computer tomography scanning and evaluated according to RECIST criteria before and after 12 weeks of treatment. Wellbeing was judged weekly by the investigator and by quality-of-life questionnaires by the patients. In view of the small number of patients, no statistical testing was performed.

**Results:**

All 12 patients enrolled had progressive, heavily pre-treated advanced breast cancer. No treatment-related serious adverse events or DLTs occurred during the first 4 weeks of E4 treatment allowing the investigation of all three doses. Five of nine patients completing 12 weeks of E4 treatment showed objective anti-tumor effects and six of nine patients reported improved wellbeing.

**Conclusion:**

High doses of estetrol seem to be safe and are well tolerated during 12 weeks of treatment without dose-limiting toxicity and with anti-tumor effects in five of nine heavily treated patients with progressive, anti-estrogen resistant, advanced breast cancer.

## Introduction

Breast cancer is the most commonly diagnosed cancer and the leading cause of cancer death in women (Bray et al. 2018), with metastatic disease accounting for the majority of deaths (Dillekas et al. 2019). In most women treated with anti-estrogens, the mainstay of initial treatment, recurrence due to the development of drug resistance occurs, especially in the metastatic setting (D'Souza et al. 2018; Haque and Desai 2019; Szostakowska et al. 2019). Resistance to endocrine therapy is a major challenge, prompting the need for new treatment options (D'Souza et al. 2018; Haque and Desai 2019; Szostakowska et al. 2019). In particular, there is a need for efficacious treatments that can improve or maintain patients’ quality of life (QoL) (Cardoso et al. 2014; Janni et al. 2019).

High doses of estrogens (HDE), such as ethinylestradiol (EE), estradiol (E2) and diethylstilbestrol (DES), are effective for the treatment of breast cancer, especially in postmenopausal women, who are at least 5 years after menopause and/or in women who are resistant to anti-estrogens (Coelingh Bennink et al. 2017a). Due to side effects, especially thrombosis and other cardiovascular complications, HDE treatment has been replaced by anti-estrogens, such as tamoxifen, aromatase inhibitors (AIs) and fulvestrant (Croxtall and McKeage 2011; Ingle et al. 1981; Reinert and Barrios 2017). However, due to their strong anti-estrogenicity, these compounds induce serious unwanted signs and symptoms of estrogen deficiency, interfering with QoL and especially with long-term drug compliance (Ciruelos et al. 2014; Kwan et al. 2017; Makubate et al. 2013). Recently, CDK4/6 inhibitors have obtained an important position in the treatment of advanced breast cancer after failure of anti-estrogens, but these compounds also have side effects and interfere with QoL (Howie et al. 2019; Lasheen et al. 2017; Messina et al. 2018).

**Fig. 1:**
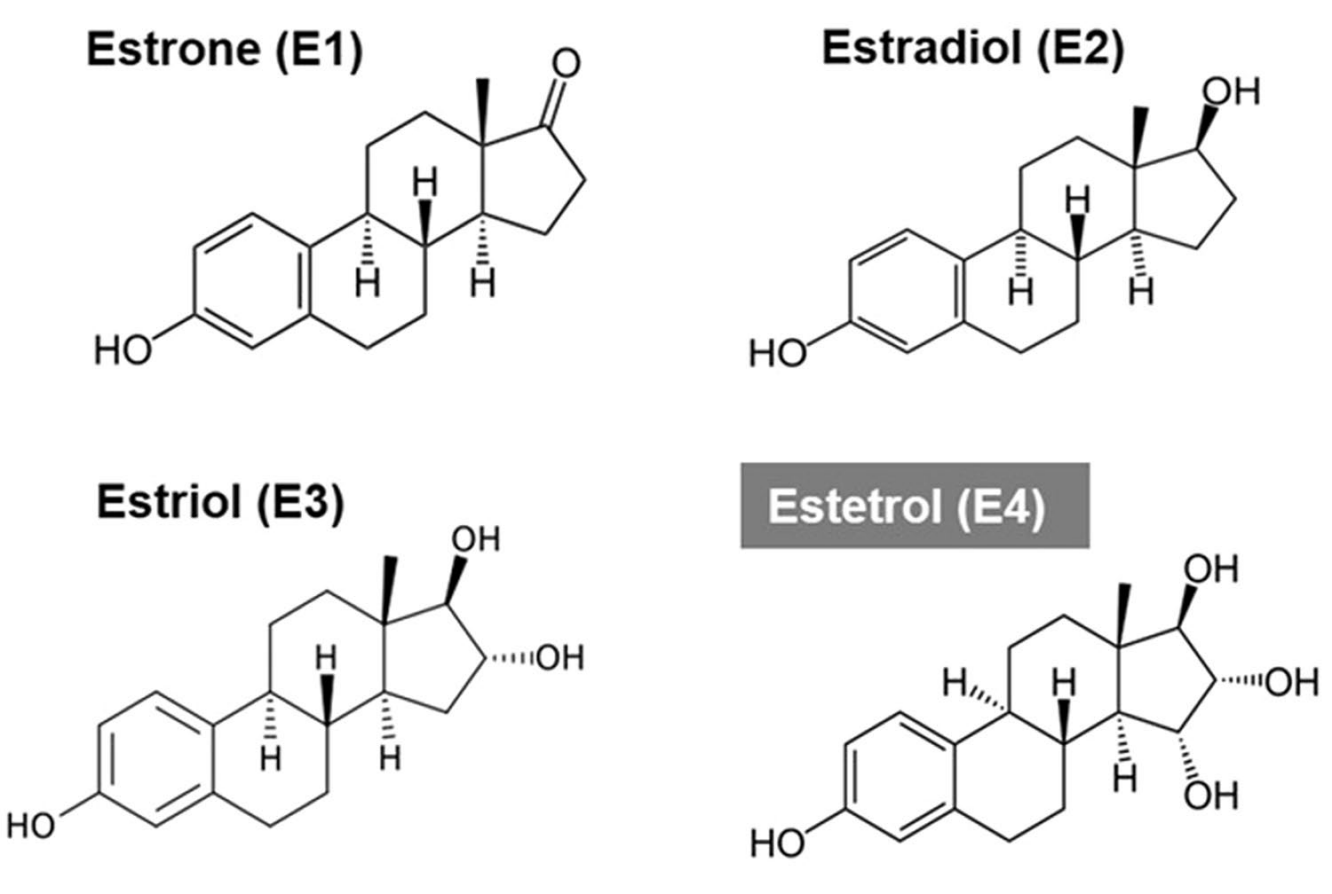
The four natural estrogens

The fetal estrogen estetrol (E4) is a potential new HDE treatment for anti-estrogen-resistant ER+/HER− advanced breast cancer. The term “estetrol” refers to estra-1,3,5(10)-triene-3,15α,16α,17β-tetrol, an estrogenic steroid, produced under physiological conditions only during human pregnancy by the fetoplacental unit (Hagen et al. 1965). It is also known as E4, referring to the four OH groups in the molecule at positions 3, 15, 16 and 17 (Fig. 1). Estetrol was first identified by Egon Diczfalusy et al. in 1965 at the Karolinska Institute in Stockholm, Sweden (Hagen et al. 1965) and was discovered as a potential estrogenic drug for human use in 2001 by the last author of this paper at Pantarhei Bioscience in the Netherlands (Hagen et al. 1965; Holinka et al. 2008). Extensive (human) pharmacological and safety and dose-finding studies have been performed for the development of E4 as the estrogen in a combined oral contraceptive (COC) and for menopausal hormone therapy (MHT) (Apter et al. 2016; Coelingh Bennink et al. 2008b, 2016, 2017b, c; Duijkers et al. 2015; Gaspard et al. 2020; Holinka et al. 2008). These studies have demonstrated a more favorable cardiovascular risk profile of E4 compared to other natural and synthetic estrogens used for these applications in the past (Apter et al. 2016; Coelingh Bennink et al. 2016). For both the COC and the MHT application, a daily dose of 15 mg E4 has been selected and studied in randomized controlled studies (Apter et al. 2016; Gaspard et al. 2020).

There are several reasons why E4 in particular can be used for a HDE in cancer treatment. First, E4 has a long oral elimination half-life (Visser et al. 2008), which makes it suitable for use as a once-a-day oral drug. Second, it is a metabolic end product, without active and potentially toxic metabolites (Coelingh Bennink et al. 2008a). Third, compared to other estrogens, E4 shows little interaction with liver function and steroid- and drug-metabolizing liver enzymes (Mawet et al. 2015). In particular, the limited interference of E4 with liver factors involved in hemostasis provides hope for a lower risk of cardiovascular (CV) side effects (Douxfils et al. 2020; Kluft et al. 2017), which was the major reason for discarding estrogens for the treatment of breast cancer in the past. Fourth, in preclinical breast cancer studies, in in vitro models, E4 antagonized the proliferative effect on tumor growth of E2 in MCF-7 and LTED cells (Giretti et al. 2014). Fifth, in the in vivo rat DMBA model, high-dose E4 completely prevented breast tumor development comparable to ovariectomy, whereas in the therapeutic DMBA model, E4 inhibited the further growth of existing mammary tumors (Visser et al. 2012). Last but not least, high-dose E4 (HDE4) tumor treatment is expected to improve patients’ wellbeing and QoL by decreasing signs and symptoms of estrogen deficiency due to postmenopausal or anti-estrogenic estrogen depletion.

The first study with E4 in women with breast cancer was performed by Singer et al. in 30 women with recently diagnosed ER+ breast cancer, who were treated in a prospective, randomized, double-blind, placebo-controlled, neo-adjuvant study for 2 weeks with 20 mg E4 per day or placebo. The most relevant results of this study were that E4 significantly induced apoptosis and increased the expression of the anti-proliferative ERbeta receptor in the breast tumors (Singer et al. 2014).

Altogether, the data suggest that: (1) E4 may be safer for the CV system than other estrogens, (2) HDE4 may be an effective anti-tumor treatment and (3) HDE4 may maintain or improve QoL.

Here, we report the results of the “Advanced Breast Cancer Estetrol” (ABCE4) study, a phase IB/IIA, dose-escalation study with E4 in postmenopausal women with progressive ER+/HER2− end-stage breast cancer with resistance or intolerance to tamoxifen or AIs without established therapeutic alternatives, including chemotherapy. The primary objective of this study was to assess the dose-limiting toxicity (DLT) and to estimate the maximum recommended and optimal dose of E4. The secondary objectives included the assessment of the safety and tolerability of E4, the subjective clinical evaluation of QoL related to estrogen-deficiency symptoms, and a preliminary assessment of the efficacy of HDE4 in terms of anti-tumor response.

## Methods

### Study design and patients

The ABCE4 study was an open-label, phase IB/IIA, dose-escalation study performed in two centers in Germany (ClinicalTrials.gov identifier: NCT02718144; Eudra-CT number 2016-003707-57). It consisted of a 3 + 3 cohort design, whereby successive cohorts of three patients received 20 mg, 40 mg, or 60 mg E4 per day by oral administration.

Key inclusion criteria included: a natural or surgical menopause at least 5 years previously; progressive ER+ and HER2− locally advanced and/or metastatic breast cancer without established therapeutic alternatives; failure of anti-estrogen treatment with tamoxifen and/or AI(s) due to the development of resistance or unacceptable side effects; a life expectancy of at least 6 months and a body mass index (BMI) between 18 and 35 kg/m^2^. Patients with a history of venous or arterial thromboembolic disease or a known defect in the blood coagulation system were excluded. In addition, patients who had been treated with fulvestrant within 6 months prior to the start of E4 treatment were excluded, since this treatment destroys the ER, which is essential for allowing E4 receptor-mediated efficacy. As of the second cohort, patients with a history of severe cardiac events or life-threatening cardiac dysrhythmia, unstable angina or clinical congestive heart failure were excluded and patients had to have an Eastern Cooperative Oncology Group (ECOG) performance status of 0–2.

### Ethics

The study was conducted according to Good Clinical Practice (GCP) and in accordance with the Declaration of Helsinki and International Council for Harmonization of Technical Requirements for Pharmaceuticals for Human Use. The study was approved by the independent ethics committee Landesärztekammer Rheinland-Pfalz (Germany). Each patient provided written informed consent prior to screening. An independent data monitoring committee was in place to oversee the study, to evaluate safety and to decide on dose escalation.

### Study medication

Patients were treated for 12 weeks with E4; the first 4 weeks in a phase IB safety setting and thereafter 8 weeks in phase IIA to extend the safety observations and allow for a preliminary efficacy evaluation. Phase IB of the study followed the traditional 3 + 3 study design to determine the optimal dose of E4. Patients were treated in cohorts of 3 receiving the same dose. Occurrence of DLTs at completion of phase IB after 4 weeks treatment determined escalation to the next higher dose. After completion of the 20 mg and the 40 mg E4 dose cohort, safety and tolerance data were evaluated before proceeding to the next higher dose.

After completion of phase IB, patients continued treatment for 8 weeks at their individual phase IB dose level to assess the safety and preliminary anti-tumor response in phase IIA. Thereafter, further extension of treatment was possible beyond 12 weeks.

The study medication was supplied in tablets of 20 mg for oral administration and packed in blisters by Haupt Pharma, Münster, Germany. All tablets (1, 2 or 3) were to be taken once daily in the morning.

### Study procedures

Patients were screened before the start of study medication. Study visits were scheduled on Days 1, 8, 15, 28, 56, and 84. In addition, patients were contacted by telephone on Days 42 and 70 for an additional check of wellbeing. Safety, tolerance and toxicity assessments included the recording of adverse events (AEs) using Common Terminology Criteria for Adverse Events, version 4.03 (CTCAE v4.03), physical examination, vital signs (blood pressure and heart rate), body weight, electrocardiograms and safety laboratory parameters (hematology and biochemistry). Treatment-emergent adverse events (TEAEs) were defined as those AEs occurring from the time of first study medication intake until the last visit.

Objective anti-tumor effects were assessed by CT scanning and evaluated according to the RECIST criteria (version 1.1) before and after 12 weeks of treatment and thereafter every 12 weeks in case of extended treatment. External independent verification of the CT scans was performed by Prof Adrian Lim, Clinical Radiologist, Imperial College, London, UK. Wellbeing (QoL) was assessed by means of specific questions about estrogen-deficiency symptoms, derived from the validated Functional Assessment of Cancer Therapy Endocrine Subscale (FACT-ES) (Fallowfield et al. 1999). A safety review committee (SRC) including the investigators and sponsor representatives had weekly meetings to evaluate the safety and tolerability of E4 continuously and to assess wellbeing of the patients based on the clinical judgement of the treating oncologists [MS and AH]. At the end of the treatment, patients completed a treatment satisfaction questionnaire.

### Statistical analysis

This was a dose-escalation study, with no formal hypothesis testing, and hence no sample size calculation was performed. In view of the small numbers of study participants required for this standard 3 + 3 design study, no statistical test procedures have been performed and individual results are presented without means and standard deviations or medians and ranges.

All subjects who received treatment were included in the evaluation of the safety data. The per-protocol population, defined as the subjects who completed the study without major protocol deviations, was used for the evaluation of efficacy.

## Results

### Patients

**Fig. 2:**
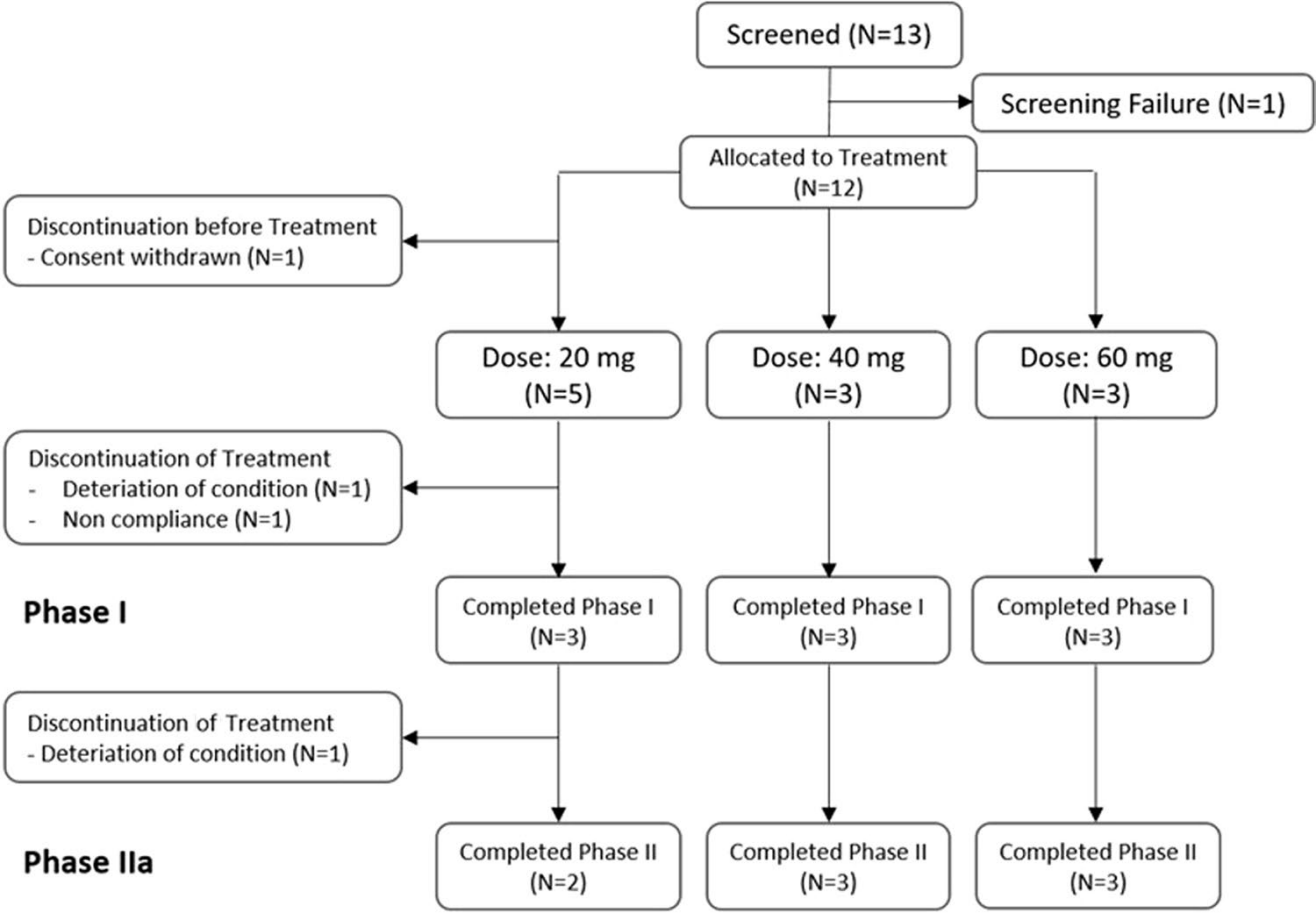
Patient disposition (CONSORT diagram)

A total of 12 postmenopausal women with heavily pretreated breast cancer were enrolled in the ABCE4 study, of whom 11 received treatment (Fig. 2). One patient withdrew informed consent prior to starting treatment. Nine patients completed phase IB. Two patients withdrew in phase IB due to the severity of their disease (disease progression and non-compliance due to the severity of disease).

Eight patients completed both the phase IB and IIA part of the study. One patient in the 20 mg group discontinued the study during phase IIA due to disease progression after 9.5 weeks of E4 treatment. She died 2 weeks later.

Six patients requested continuation of E4 treatment beyond the trial period of 12 weeks, with the other two patients stopping at the end of phase IIB period due to disease progression. Overall, there were no discontinuations due to drug-related adverse events during the phase IA/IIB period.

At the data cut-off date for this paper (November 2020), one patient was still continuing treatment with 20 mg E4 (35 months of treatment). During these almost three years of treatment with 20 mg E4 with stable BC disease, this patient experienced three episodes of vaginal bleeding after 7, 9 and 19 months and of 1, 9 and 1 day duration, respectively. A D&C was performed during the second and third events. No endometrial abnormalities were found. The other five patients requesting follow-up E4 treatment discontinued after 13–48 weeks of treatment due to progression of the BC disease. In two patients taking 20 and 40 mg E4, a short and mild episode of vaginal bleeding was reported after 5 and 4 months treatment, respectively, not requiring intervention and with spontaneous recovery.

### Baseline characteristics

Table 1 provides the baseline patient characteristics, the details concerning the breast cancer status and the medical and treatment history of the 11 patients treated. All had experienced a spontaneous or medically induced menopause more than 5 years ago and had been treated with anti-estrogenic drugs until resistance occurred. All patients had been treated with multiple endocrine, targeted and chemotherapies except one patient (no. 2), who had refused further targeted or chemotherapy after resistance to endocrine therapy had occurred. This is the patient still on 20 mg E4 treatment at the time of writing this manuscript.

**Table 1:** Demographics and baseline disease characteristics

Parameter	Cohort 20 mg E4					Cohort 40 mg E4			Cohort 60 mg E4		
Patient	1^b^	**2**	3^a^	4^a^	**5**	**6**	**7**	**8**	**9**	10	11
Age, yrs	56	72	77	70	78	68	78	79	65	69	66
Weight, kg	70	54	72	76	52	43	58	82	61	58	63
BMI, kg/m^2^	25	22	27	27	20	16	23	28	20	23	23
Duration since last natural menses, yrs	8	19	40	18	33	13	31	30	14	20	20
Duration since BC diagnosis, yrs	8	5	13	–	37	23	13	16	8	16	20
Hormone receptor status	ER^+^/PR^+^/HER2−	ER^+/^PR^+^/HER2−	ER^+^/PR^+^/HER2−	ER^+^/PR^+^/HER2−	ER^+^/PR^+^/HER2−	ER^+^/PR^−^/HER2−	ER^+^/PR^+^/HER2−	ER^+^/PR^+^/HER2−	ER^+^/PR^−^/HER2−	ER^+^/PR^+^/HER2−	ER^+^/PR^+^/HER2−
TNM stage	IV	IV	IV	IV	IV	IV	IV	IV	IV	IV	IV
ECOG status	1	0	0	3	0	0	0	1	0	1	1
Metastases	Bo, Li, LN	Bo	Bo, Li, LN, Lu	Bo, Br, Li, Lu	LN, Lu	Lu	Li	Bo, LN, Lu	Li, Bo	Li	Bo, Li, LN, Lu
Notable complications	Ma, Pe	None	Pe	Pe	None	Pe	None	None	None	None	Pm
Prior BC treatment, n^c^											
ET	2	3	3	5	2	5	4	3	5	2	4
Chemotherapy	3	–	2	1	–	4	3	2	5	3	5
TT	1	–	–	–	–	1	1	–	1	–	–
ET + TT	1	–	–	1	2	1	–	–	–	2	1

All women were at least 5 years postmenopausal; data are shown per patient in each cohort; Bold patient numbers indicate women who entered the follow-up period

*BC* breast cancer, *Bo* bone, *Br* brain, *E4* estetrol, *ER* estrogen receptor, *ET* endocrine therapy, *HER2−* human epidermal growth factor receptor 2 negative, *Li* liver, *LN* lymph nodes, *Lu* lung, *MA* malignant ascites, *Pe* pleural effusion, *Pm* peritoneal metastases, *PR* progesterone receptor, *TT* targeted therapy, *yrs* years

^a^Patient discontinued during phase IB

^b^Patient discontinued during Phase IIA

^c^Not including radiotherapy or surgery

### Dose-limiting toxicity, safety and tolerability

None of the patients experienced a DLT. All three E4 doses were well tolerated by all 11 patients treated. In total, 31 TEAEs were reported by 8 patients, mainly of mild or moderate intensity (Table 2). Six events were considered possibly related to E4 treatment: dry skin, pruritis and endometrial hyperplasia in one patient using 20 mg E4, fatigue and 4 days vaginal bleeding resolving spontaneously in a second patient on 20 mg E4 and regurgitation in a third patient on 40 mg E4. No drug-related serious AEs were reported.

**Table 2:** Number of patients reporting adverse events

Adverse events, *n* (%)	20 mg cohort (*N* = 5)	40 mg cohort (*N* = 3)	60 mg cohort (*N* = 3)	Total (*N* = 11)
TEAEs	5 (100)	1 (33.3)	2 (66.7)	8 (72.7)
Drug-related TEAEs	2 (40)	1 (33.3)	0	3 (27.3)
Grade 3 or 4 TEAEs	1 (20)	0	1 (33.3)	2 (18.2)
SAEs	3 (60)	0	1 (33.3)	4 (36.4)
Drug-related SAEs	0	0	0	0
TEAEs resulting in study drug discontinuation	2 (40)	0	0	2 (18.2)

*TEAEs* treatment-emergent adverse events, *SAEs* serious adverse events

### Anti-tumor activity

**Fig. 3:**
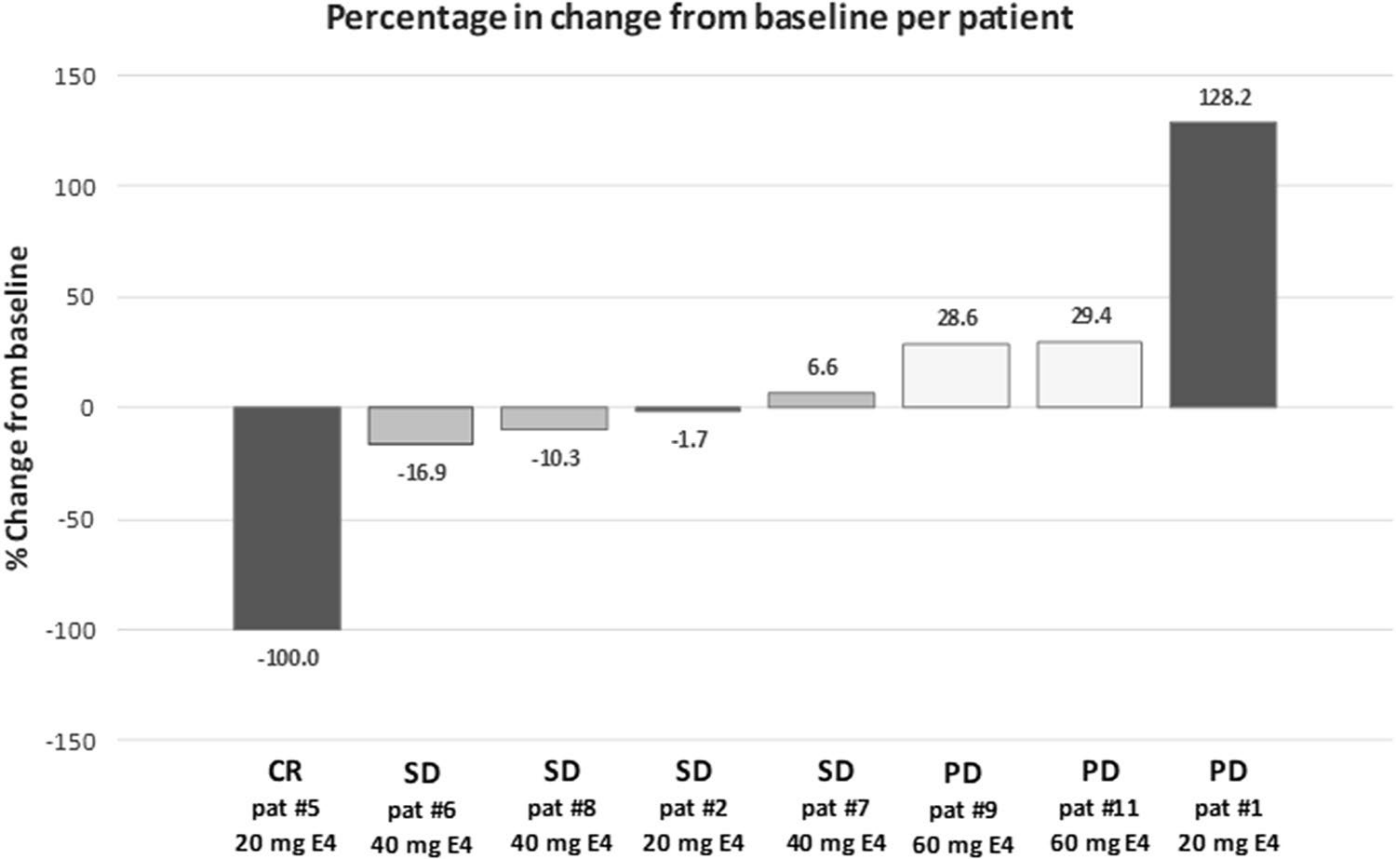
Percentage change of tumor diameters from baseline per patient

After 12 weeks of E4 treatment, five of nine patients showed objective anti-tumor effects with stable disease in four patients and one complete response, judged according to RECIST criteria (Table 3). The percentage change of tumor diameters from baseline per patient in eight patients is shown in Fig. 3. Both the complete response as well as the highest increase of tumor diameter occurred with the 20 mg E4 dose. The third patient in the 20 mg group and all 3 patients treated with 40 mg E4 showed stable disease. All three patients treated with the highest E4 dose of 60 mg demonstrated progressive tumor growth. The five patients demonstrating an anti-tumor effect requested continuation of E4 treatment beyond the trial. Tumor assessment after 24 weeks of treatment confirmed stable disease in four of four patients investigated.

**Table 3:** Anti-tumor response after 12 weeks of E4 treatment according to RECIST criteria

Evaluation	20 mg E4			40 mg E4			60 mg E4		
Patients	1	2	5	6	7	8	9	10	11
Evaluation of target lesions	PD	SD	CR	SD	SD	SD	PD	PD	PD
Evaluation of non-target lesions	PD	Non-CR/non-PD	Non-CR/non-PD	Non-CR/non-PD	Non-CR/non-PD	Non-CR/non-PD	PD	–	PD
New lesions	Yes	No	No	No	No	No	Yes	Yes	No
Response type	PD	SD	CR	SD	SD	SD	PD	PD	PD
Response	No	Yes	Yes	Yes	Yes	Yes	No	No	No

*E4* estetrol, *CR* complete response, *SD* stable disease, *PD* progressive disease

### Quality of life

**Fig. 4:**
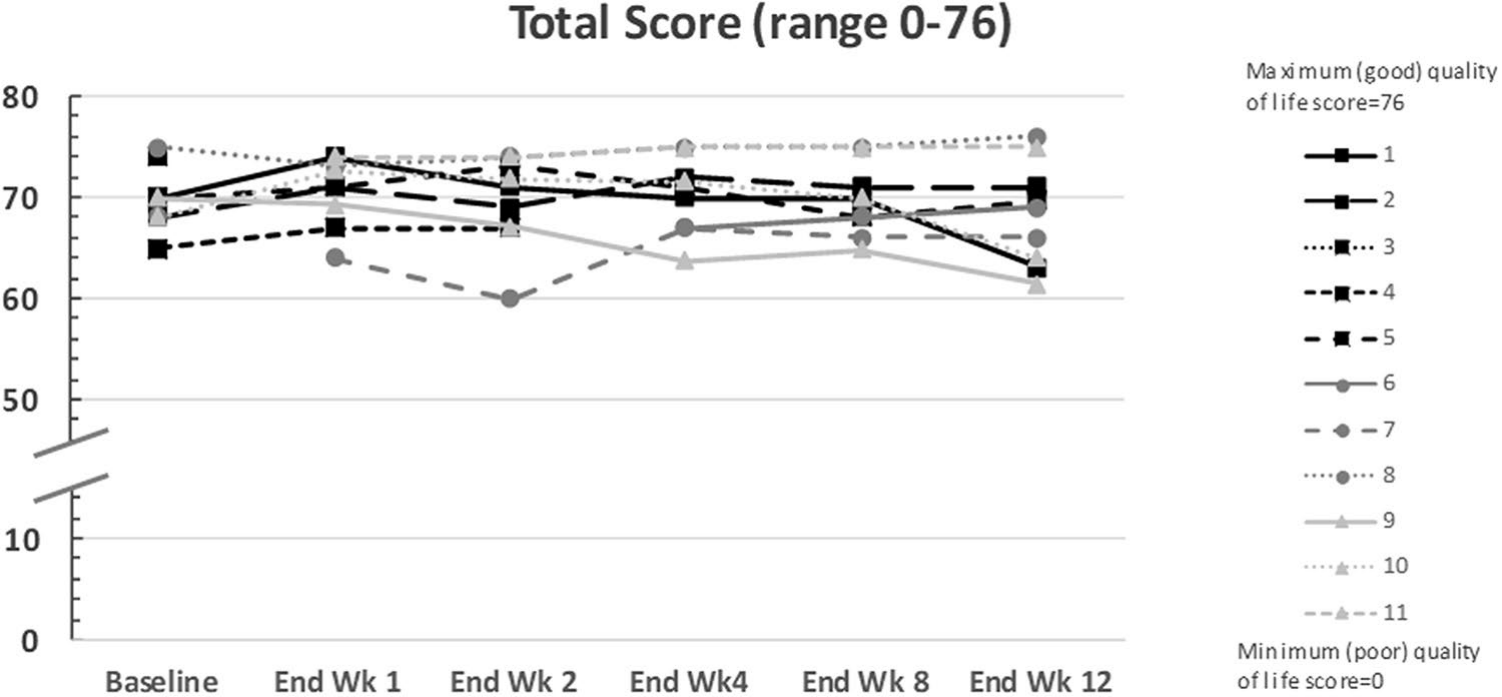
Total quality-of-life score

Overall, the total QoL scores for estrogen-deficiency symptoms in the nine treated patients were maintained at high levels from baseline to the end of the phase IIb period (range 65–75 at baseline to 61.5–76 at end of treatment; Fig. 4). Based on investigator’s reporting during the SRC meetings, six of nine patients mentioned verbally to the investigators that they felt very well. Four patients reported to be very satisfied with the medication and these patients also said they would consider taking the medication in the future, whereas one patient was very dissatisfied with the result of treatment.

## Discussion

The results of this first study with high daily doses of 20, 40 or 60 mg of the fetal estrogen estetrol (E4) in nine patients with progressive locally advanced and/or metastatic breast cancer showed no DLTs or serious adverse events related to E4 during 12 weeks of treatment and was well tolerated by all patients. High daily doses of 20 or 40 mg E4 demonstrated a significant anti-tumor effect in five of nine patients after 12 weeks of E4 treatment with disappearance of some tumors and no further growth of other tumors. According to the RECIST criteria, the effect of HDE4 was classified as stable disease in four cases and a complete response in one case. These results are consistent with earlier in vivo observations in the DMBA rat model mentioned in the introduction of this paper (Visser et al. 2012).

From the 1940s to the 1960s, HDE treatment was routinely used for the endocrine treatment of advanced breast cancer and was later replaced by tamoxifen, AIs and fulvestrant (Coelingh Bennink et al. 2017a). In recent years, anti-endocrine treatment has been combined with novel chemotherapeutics, such as CDK4/6 inhibitors, mTOR antagonists and PI3K-inhibitors, to overcome endocrine resistance and to delay progression of disease. All these compounds also cause side effects in addition to the known side effects of anti-estrogenic therapy.

Evidence suggests that HDE is especially effective in women with ER+ breast cancer, who are at least 5 years after menopause and/or in women who have become resistant to anti-estrogens (Coelingh Bennink et al. 2017a). The mechanism of action of HDE under these conditions has been studied extensively by the groups of Santen (Song et al. 2001) and Jordan (Jordan 2015), concluding that apoptosis was observed in responding breast tumors. This is confirmed by the first ever study with HDE4 by Singer et al. (2014), mentioned in the introduction of this paper. The molecular mechanism of action of this estrogen-induced apoptosis requires further investigation.

There is a place for a HDE treatment in patients with anti-estrogen-resistant advanced breast cancer, provided such a treatment has a more favorable CV risk profile compared to the natural and synthetic estrogens used in the past. Preclinical and clinical studies with E4 support the potentially favorable CV safety profile of E4 in dosages up to 15 mg E4 per day for a year in contraceptive studies (Kluft et al. 2017; Mawet et al. 2015), and up to 40 mg E4 per day for 28 days in MHT dose-finding studies (Coelingh Bennink et al. 2016).

Based on preclinical and clinical contraceptive and menopausal studies, the potency of E4 in the human is about 10× lower than that of E2 (Coelingh Bennink et al. 2016; Duijkers et al. 2015), so the dose of 20, 40 and 60 mg E4 used by the patients reported here, is comparable to 2, 4 and 6 mg E2. Ellis et al. investigated the effect of high-dose 6 mg and 30 mg E2 in women with anti-estrogen-resistant advanced breast cancer (Ellis et al. 2009). They observed equal efficacy of the 6 and 30 mg E2 doses, but the higher 30 mg dose showed more serious side effects and drop-outs (Ellis et al. 2009). Based on these data and the observed potency ratio between E2 and E4 of a factor 10, the maximum E4 dose in the present E4 dose-escalation study was 60 mg.

We tested E4 in heavily pretreated late-stage patients with a life expectancy sufficiently long to enable judgement of the new treatment. Therefore, the anti-tumor effect in five of nine patients treated for 12 weeks, which was continued under compassionate use in five cases for 13–48 weeks, with one patient still on treatment to date (November 2020), is rather impressive, taking into consideration the seriousness of their disease.

These end-stage disease women with breast cancer are generally seriously estrogen deficient for very long periods, either by spontaneous or by induced menopause or more often due to long-term anti-estrogen treatments. This may cause not only annoying hot flushes and sweating interfering with sleep, but also osteoarthralgia (Fenton and Panay 2016). Furthermore, depression, and other mood changes, as well as more objective signs of estrogen deficiency, such as bone loss, fractures and cognition problems, occur. These signs and symptoms of estrogen deficiency are an important cause of non-compliance to and early discontinuation of anti-estrogen treatment. The use of HDE4 is expected to improve drug compliance and increase wellbeing, thereby creating a dual high-dose E4 efficacy of (1) an anti-tumor effect and (2) strong estrogen substitution. Short and spontaneously resolving vaginal bleeding suggesting some endometrial interaction occurred in the study once and during limited duration follow-up in two patients. The patient treated for almost 3 years now, with 20 mg E4 per day experienced three episodes of vaginal bleeding with no signs of endometrial hyperplasia after D&C. Nevertheless, HDE4 treatment of advanced BC in women with a uterus requires some type of endometrial control, although the results of this study are reassuring and suggest little effect of HDE4 on the endometrium. Rather surprisingly, the questionnaire for estrogen deficiency used in this study did not reveal estrogen-deficiency complaints at baseline. This may be due to the stage of the disease with low priority for not life-threatening complaints. According to the QoL questionnaire, quality of life was maintained by all nine patients treated. Six of nine patients reported improved wellbeing during treatment to the oncologist and after treatment four were very satisfied and another four were satisfied by the HDE4 treatment. Further clinical studies are needed to confirm the safety and the extent and duration of the anti-tumor effect of E4 in larger numbers of less seriously ill breast cancer patients and to document objectively the subjective effect of the strong estrogen substitution on QoL at an earlier stage of the disease.

## Conclusion

High doses of estetrol seem to be safe and well tolerated during 12 weeks of treatment without dose-limiting toxicity and with anti-tumor effects in five of nine heavily pretreated patients with progressive, anti-estrogen resistant, advanced breast cancer.
